# Supplementary material for: Exploiting Enzyme in the Polymer Synthesis for a Remarkable Increase in Thermal Conductivity
Source: Int J Mol Sci. 2023 Apr 20;24(8):7606. doi: 10.3390/ijms24087606 (PMC10143580; doi:10.3390/ijms24087606)
Supplement: Supplementary file 1 [file ijms-24-07606-s001.zip › ijms-2319303-supplementary.pdf]

Supplementary Materials

Section A: NMR spectroscopy of polymers

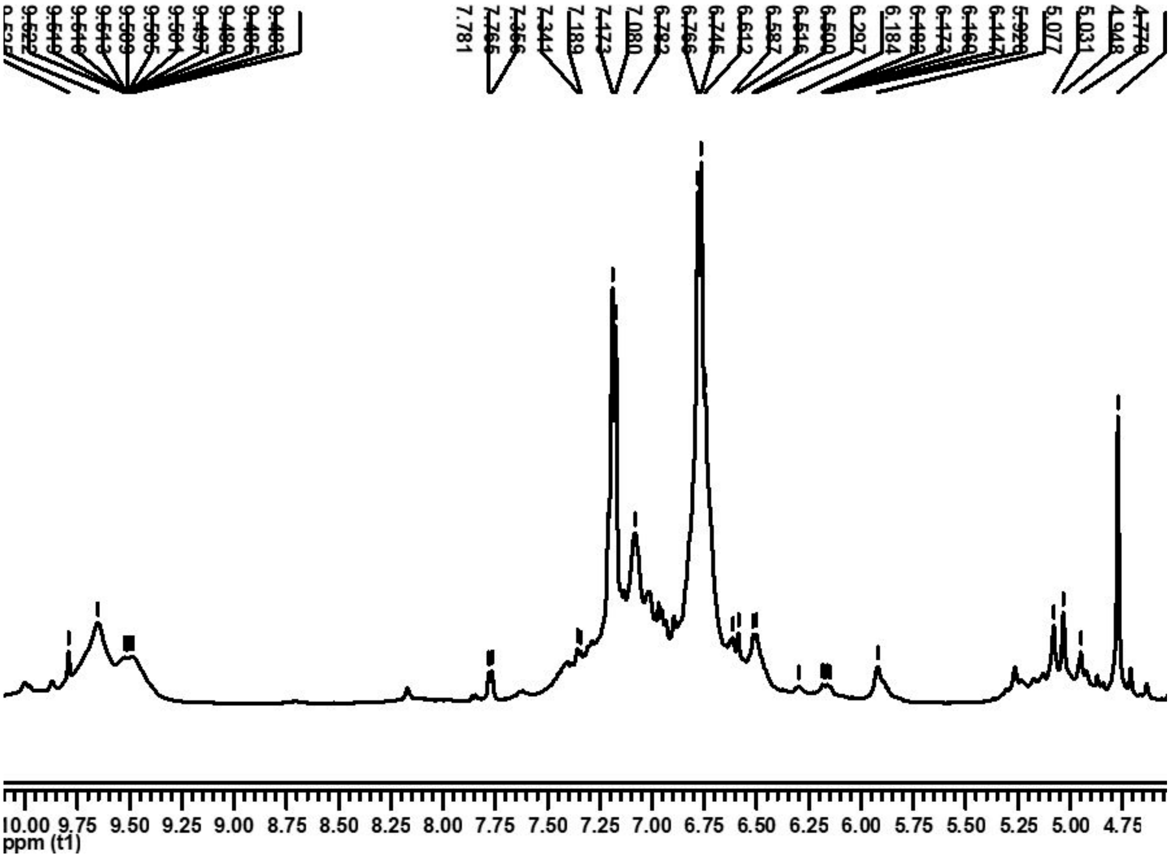

Figure S1. ^{1}H-NMR spectrum of **PBAAA_Enz** in DMSO-d6

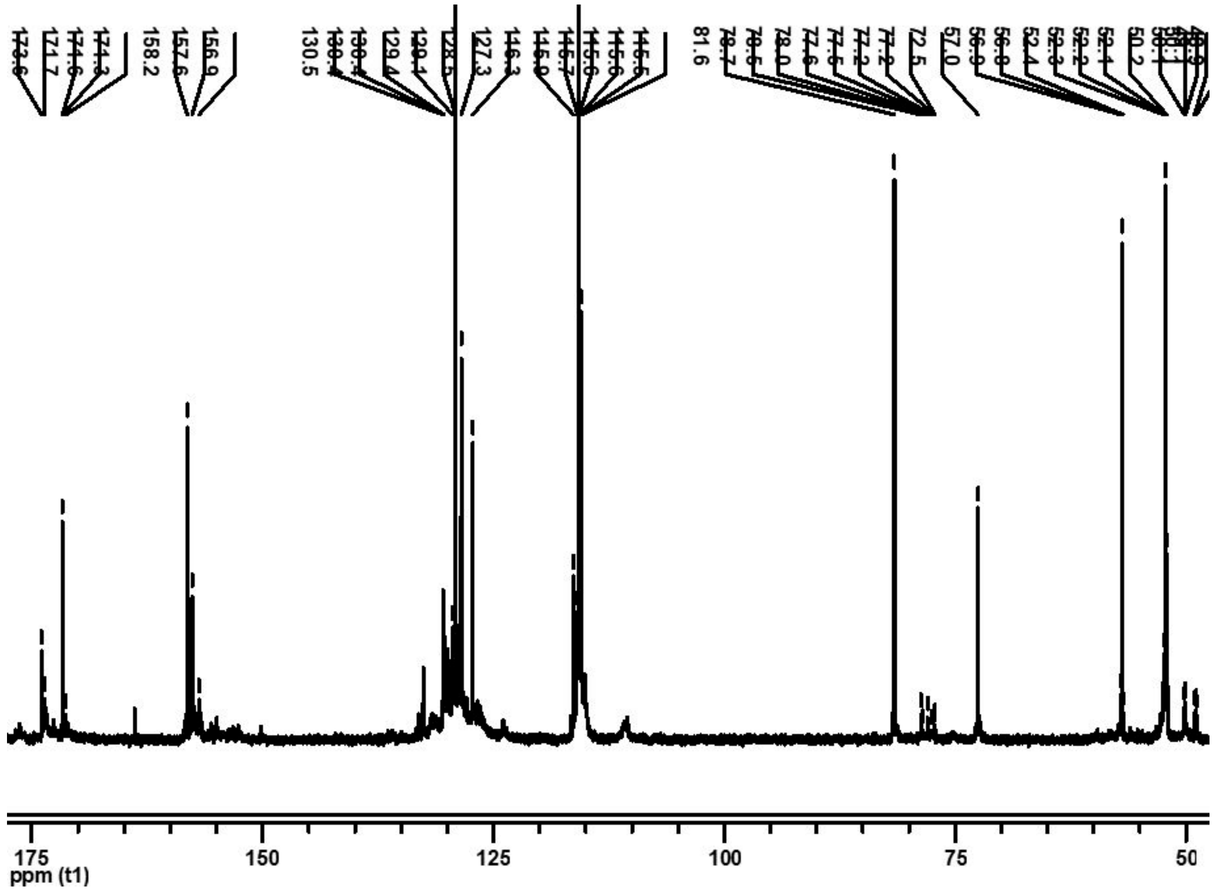

Figure S2. ^{13}C-NMR spectrum of **PBAAA_Enz** in DMSO-d6

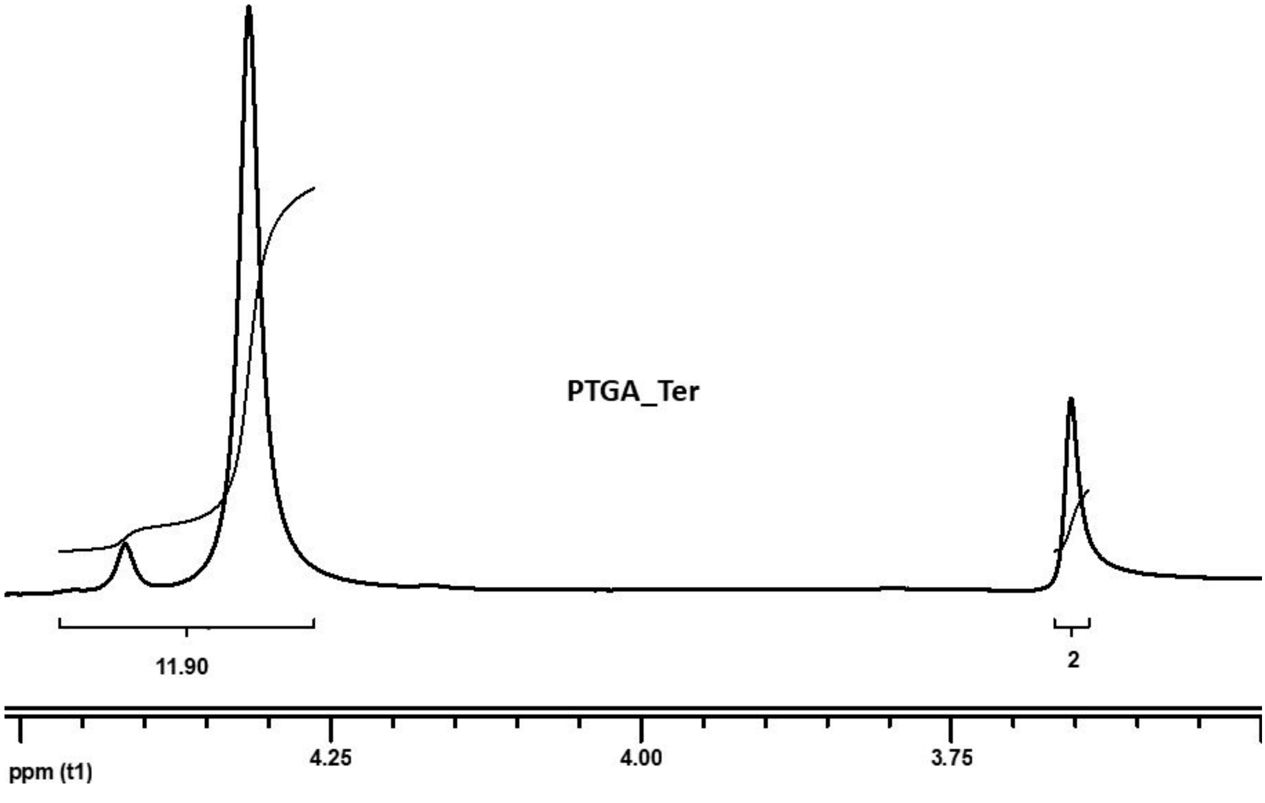

Figure S3. ^{1}H-NMR spectrum of **PTGA_Ter** in CD_3OD

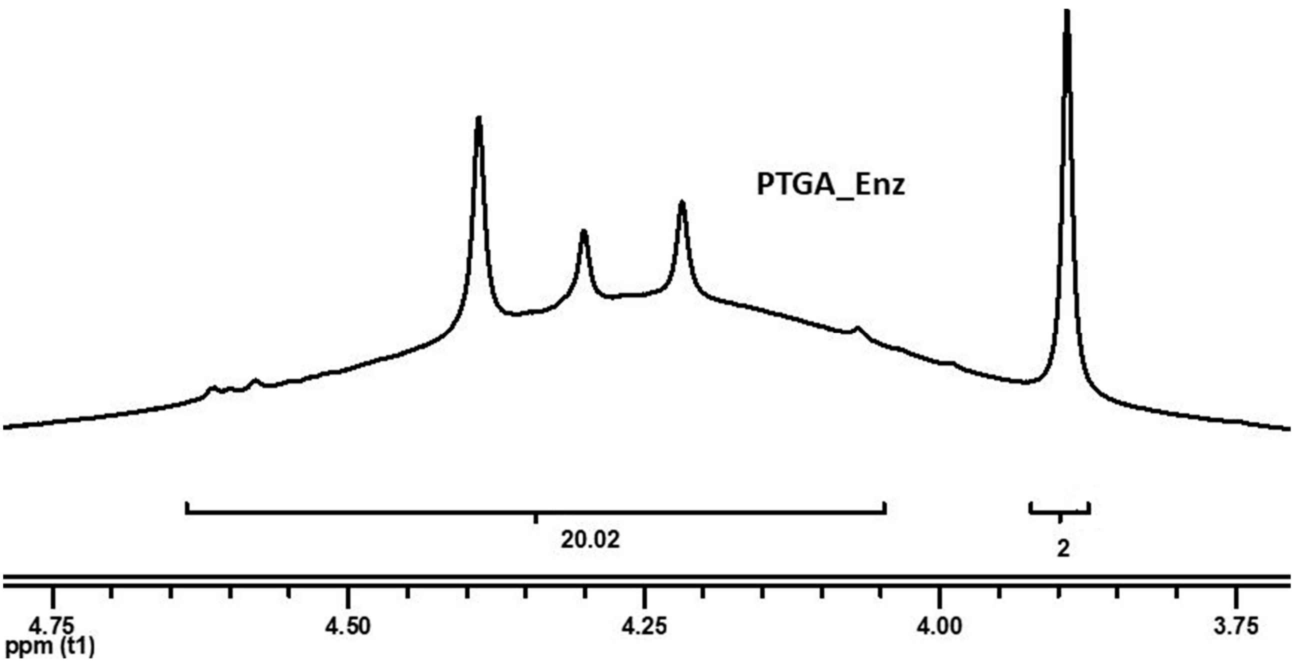

Figure S4. ^{1}H-NMR spectrum of **PTGA_Enz** in CD_3OD

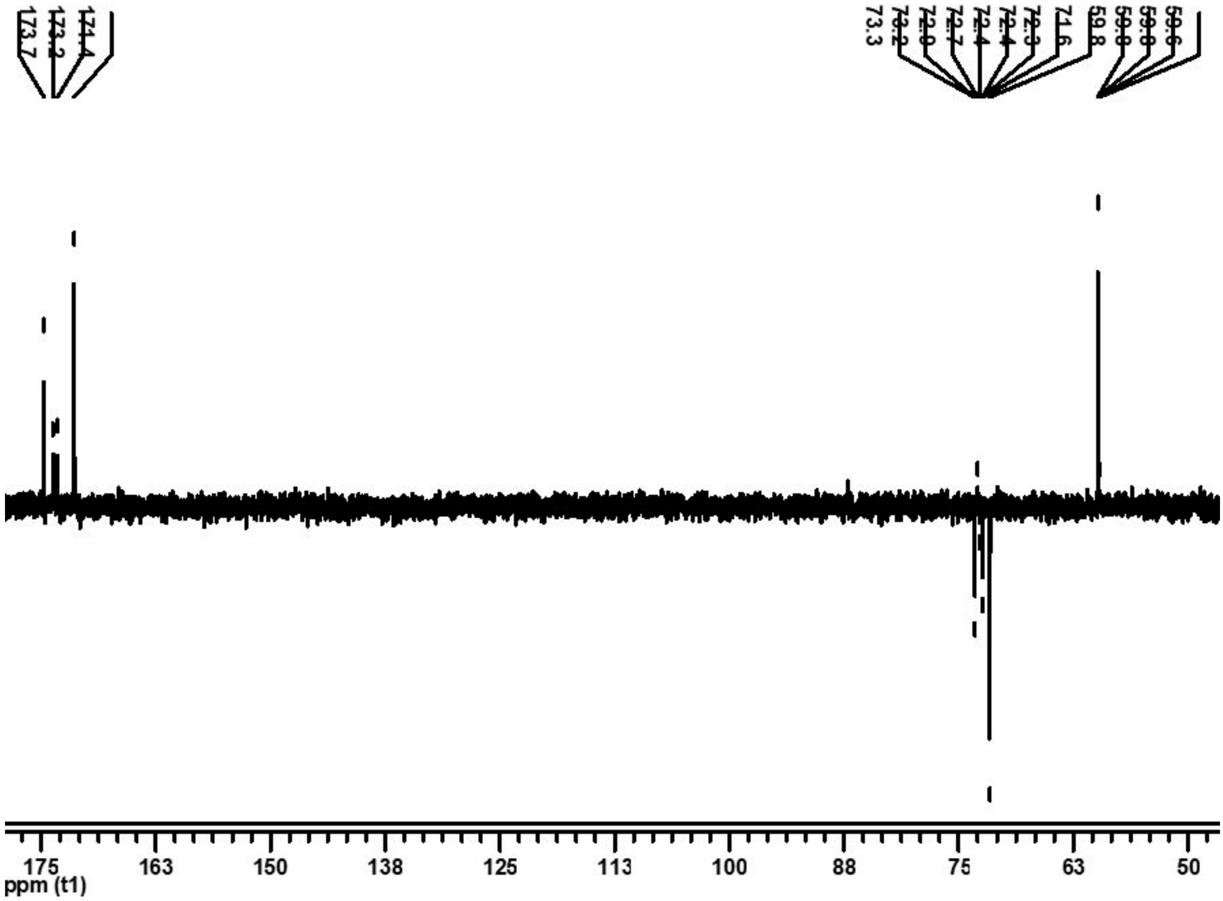

Figure S5. APT ^{13}C-NMR spectrum of **PTGA_Enz** in CD_3OD

Section B: *FTIR spectrocopy of the polymers*

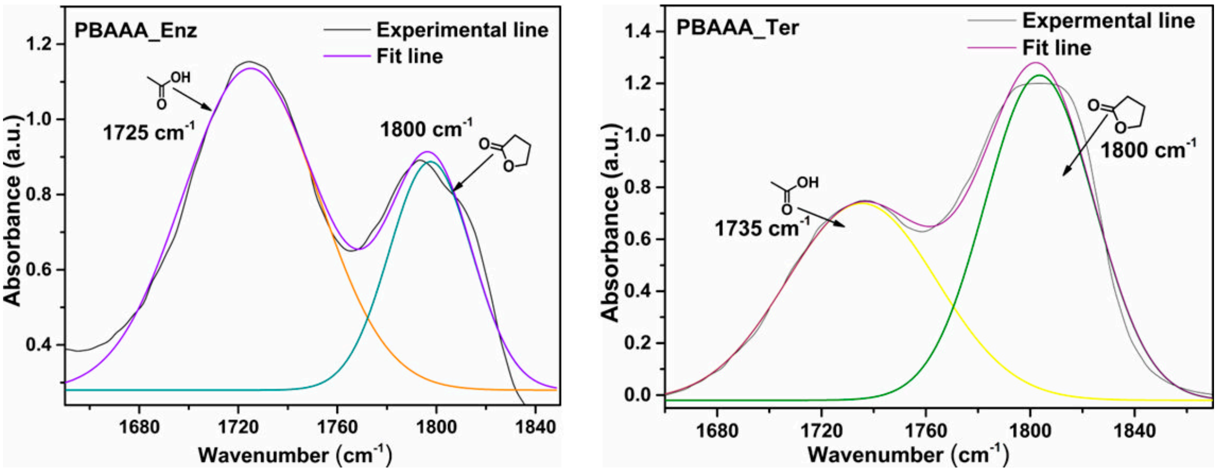

Figure S6. Structure analysis of polymer **PBAAA_Enz** and **PBAAA_Ter** by FTIR spectroscopy, the C=O band deconvolution.

Section C: *Thermogravimetric analyses of polymers*

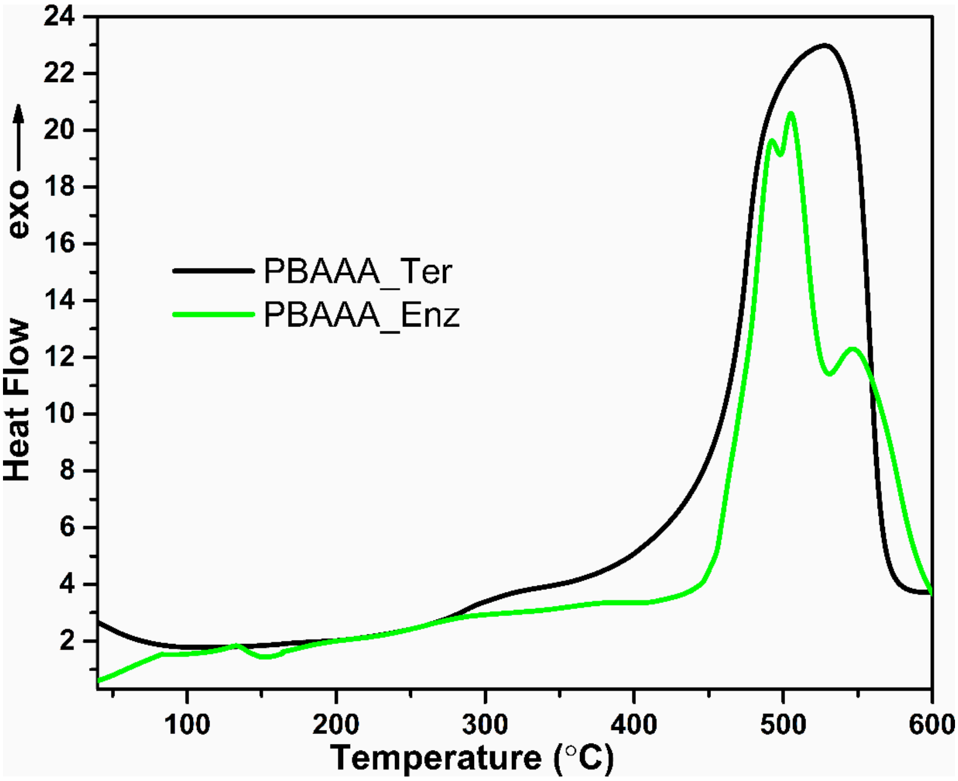

Figure S7. DTA pattern in the air of **PBAAA_Ter** and **PBAAA_Enz** at a heating rate of 10 °C min^{-1} between 30 °C and 800 °C

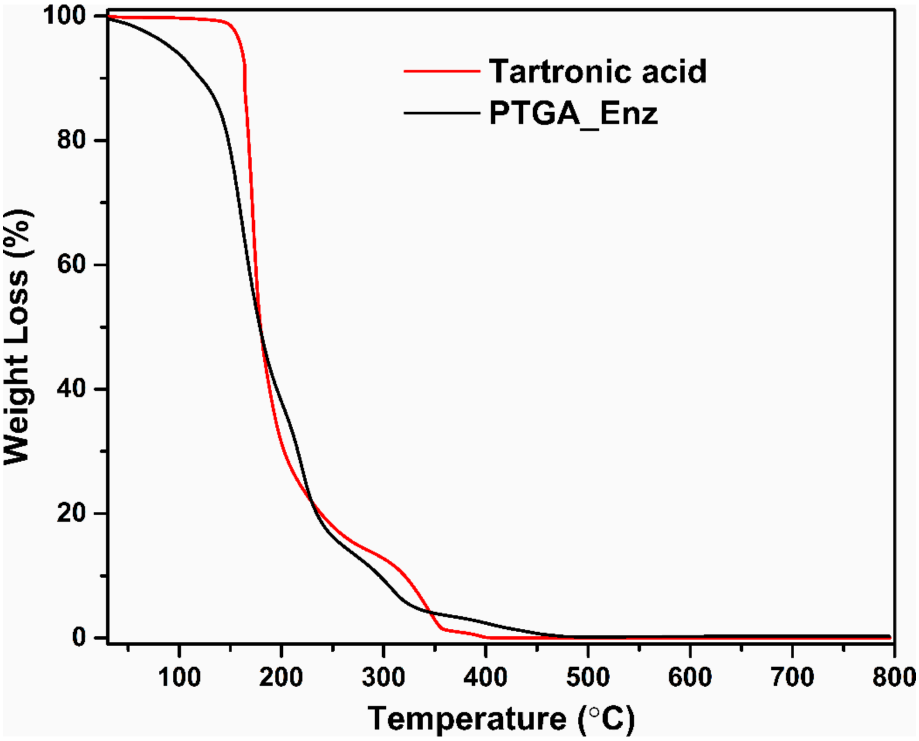

Figure S8. TGA curve of the tartronic acid and **PTGA_Enz** performed in air at a heating rate of 10 °C min^{-1} between 30 °C and 800 °C
